# The Effects of Serum Removal on Gene Expression and Morphological Plasticity Markers in Differentiated SH-SY5Y Cells

**DOI:** 10.1007/s10571-021-01062-x

**Published:** 2021-03-03

**Authors:** Alix C. Thomson, Teresa Schuhmann, Tom A. de Graaf, Alexander T. Sack, Bart P. F. Rutten, Gunter Kenis

**Affiliations:** 1 Section Brain Stimulation and Cognition, Department of Cognitive Neuroscience, Faculty of Psychology and Neuroscience, Maastricht University, Oxfordlaan 55, Maastricht, The Netherlands; 2 Department of Psychiatry and Neuropsychology, School for Mental Health and Neuroscience (MHeNS), Brain+Nerve Centre, Maastricht University Medical Centre+ (MUMC+), Maastricht, The Netherlands; 3 Maastricht Brain Imaging Centre (MBIC), Maastricht University, Maastricht, The Netherlands; 4 Centre for Integrative Neuroscience, Faculty of Psychology and Neuroscience, Faculty of Health, Medicine and Life Sciences, Maastricht University, Maastricht, The Netherlands

**Keywords:** SH-SY5Y cells, Serum deprivation, Plasticity, Human neuron model

## Abstract

Despite the widespread use of the SH-SY5Y human neuroblastoma cell line in modeling human neurons *in vitro*, protocols for growth, differentiation and experimentation differ considerably across the literature. Many studies fully differentiate SH-SY5Y cells before experimentation, to investigate plasticity measures in a mature, human neuronal-like cell model. Prior to experimentation, serum is often removed from cell culture media, to arrest the cell growth cycle and synchronize cells. However, the exact effect of this serum removal before experimentation on mature, differentiated SH-SY5Y cells has not yet been described. In studies using differentiated SH-SY5Y cells, any effect of serum removal on plasticity markers may influence results. The aim of the current study was to systematically characterize, in differentiated, neuronal-like SH-SY5Y cells, the potentially confounding effects of complete serum removal in terms of morphological and gene expression markers of plasticity. We measured changes in commonly used morphological markers and in genes related to neuroplasticity and synaptogenesis, particularly in the BDNF-TrkB signaling pathway. We found that complete serum removal from already differentiated SH-SY5Y cells increases neurite length, neurite branching, and the proportion of cells with a primary neurite, as well as proportion of βIII-Tubulin and MAP2 expressing cells. Gene expression results also indicate increased expression of *PSD95* and *NTRK2* expression 24 h after serum removal. We conclude that serum deprivation in differentiated SH-SY5Y cells affects morphology and gene expression and can potentially confound plasticity-related outcome measures, having significant implications for experimental design in studies using differentiated SH-SY5Y cells as a model of human neurons.

**Supplementary Information:**

The online version contains supplementary material available at 10.1007/s10571-021-01062-x.

## Introduction

SH-SY5Y cells are a human neuroblastoma-derived cell line used to model human neurons *in vitro*. The original cells were derived from a bone marrow biopsy in 1970, and were cloned to produce the neuron-like SH-SY5Y cells that are used in a wide range of research applications today (Biedler et al. 1978). These cells synthesize various neurotransmitters, express neural markers, and can be further differentiated *in vitro* to a mature human neuronal phenotype (Jahn et al. 2017; Encinas et al. 2000, 1999; Shipley et al. 2016). Once differentiated, SH-SY5Y cells express a catecholaminergic phenotype, with the potential to synthesize both dopamine and noradrenaline (Krishna et al. 2014). They can be used to study synapse modifications and functional cellular activity with live calcium imaging or electrophysiology (Santillo et al. 2014; Toselli et al. 1996; Jahn et al. 2017). They are often used as a cell model for Parkinson’s Disease (Xicoy et al. 2017), as well as Alzheimer’s Disease (Agholme et al. 2010), neuropathogenesis of viruses (Christensen et al. 2011), screening for neurotropic properties of pharmaceuticals (Henkel et al. 2008; Xu et al. 2019), neurotoxicity (De Simone et al. 2018; Forster et al. 2016), and even as a multicellular 3D culture (Cui et al. 2017; Kapalczynska et al. 2018).

With the widespread use of this cell line to study human neuron synapse activity and neuronal plasticity *in vitro*, it is important to understand the effects of cell handling, such as the removal of serum before experimental manipulation in fully differentiated cells.

SH-SY5Y cells are grown in a basic medium containing Dulbecco’s Modified Eagle’s- Medium (DMEM), glucose, antibiotics, and supplemented with 10–20% Fetal Bovine Serum (FBS)(Xicoy et al. 2017). The use of FBS in culture media to promote growth of cells and to maintain tissues *in vitro* was introduced in 1958 (Puck et al. 1958). This serum supplementation is vital for the growth and maintenance of cell lines, as it contains many crucial proteins, vitamins, hormones and growth factors important for cell survival and proliferation (van der Valk et al. 2018).

To induce differentiation of SH-SY5Y cells to a more mature neuronal phenotype, the serum concentration is commonly reduced to 1% or 3%, along with the addition of retinoic acid (Xicoy et al. 2017; Encinas et al. 2000). After 5–20 days, depending on the differentiation protocol, the cells reach their maximum differentiation state (Encinas et al. 2000; Shipley et al. 2016; Jahn et al. 2017). Prior to experimental manipulation, e.g. exposure to potential pharmaceutical compounds, serum is often completely removed from the cultures. This is done to ensure all cells are in the same growth cycle phase before manipulation (Langan and Chou 2011), and to prevent confounding effects of the myriad of proteins and other molecular factors present in serum, which differ by serum batch and therefore introduce phenotypic variations in cell cultures (van der Valk et al. 2010). Serum components may also mask certain intrinsic growth factor (e.g. brain-derived neurotrophic factor, BDNF) effects, therefore, serum may be removed to assess the effects of BDNF in the absence of external growth factors (Zainullina et al. 2019).

Despite the common practice of serum removal before experimental manipulation in already differentiated SH-SY5Y cells, the effects of removing serum from culture media on plasticity-related gene expression and morphology markers have not yet been examined. Understanding the effects of serum removal is essential in standardizing pre-experimental protocols. If serum removal has strong effects on gene and morphological markers in already differentiated cells, any effect of experimentation may be confounded.

Here, we aim to systematically characterize the effects of completely removing serum from differentiated SH-SY5Y culture media on gene expression markers of plasticity, specifically related to an important pathway in synaptic plasticity and long-term potentiation, the BDNF-TrkB signaling pathway (Kowianski et al. 2018; Minichiello 2009; Yoshii and Constantine-Paton 2010; Andero et al. 2014; Leal et al. 2017; Niculescu et al. 2018). We also investigated the effects of serum removal on cytoskeletal markers of neuron morphology by visualizing changes in MAP2 and βIII-Tubulin.

## Methods

### Cell Culture

Cells were obtained from ATCC^®^ (CRL2266™, RRID:CVCL_0019) and were maintained and expanded according to the provided protocol. For experiments, cells were not used above passage 26.

Undifferentiated cells were cultured in DMEM/ F12, GlutaMAX™ Supplement (GibcoTM, Thermo Scientific) supplemented with 10% heat inactivated Fetal Bovine Serum (FBS, Merck), 1% penicillin–streptomycin (P/S) and 1% L-Glutamate at 37 °C and 5% CO_2_, and split at 80–90% confluency.

### Differentiation

All cells were fully differentiated to a mature neuronal-like state before experimentation. For differentiation, cells were plated in 6-well culture plates (Greiner CELLSTAR®, Merck) at approximately 2.4 × 10^4^ cells per well. Serum concentration was decreased to 3% FBS three days prior to the addition of 10 µM retinoic acid (RA; Sigma-Aldrich, R2625). A stock solution of RA was prepared in dimethylsulphoxide (DMSO; Sigma-Aldrich, 41,640) at 10 mM, and stored at −20 °C until dilution in cell culture media to a final concentration of 10 µM. Starting from the day RA was added, medium with 3% FBS supplementation was replaced every two days for a total of ten days.

### Serum Deprivation

Differentiated SH-SY5Y cells were used for serum removal experiments. Medium containing 3% serum (FBS) was removed, and the cell surface was rinsed with PBS (warmed to 37 °C) to remove all remaining serum. Next, medium without supplemented serum (0% FBS), or medium with serum (3% FBS) was added for 1 h, 3 h, 6 h or 24 h. In total there were 8 different conditions; serum and no serum for each of the 4 time points (1 h, 3 h, 6 h, 24 h).

### Microscopy

Cells were grown on 12 mm glass coverslips (VWR, 631-1577) coated with 100 µg/mL Poly-L-Ornithine (Sigma, P4957) and 1 µg/mL Laminin (Sigma, L2020) and differentiated as described above.

At the specified collection time points following complete serum removal, cells were washed in PBS (warmed to 37 °C), and fixed for 10 min in cold 4% paraformaldehyde. To stop fixation, cells were washed 3 times 5 min in cold PBS and stored in PBS at 4 °C for a maximum of two days before antibody incubation. Cells were then blocked in PBS-Tween 20, prepared with 0.2% Tween-20, and 10% donkey serum. Antibodies for visualizing neurite outgrowth (βIII-Tubulin; Cell Signaling, Cat #5568S, RRID:AB_10694505), and dendrites (MAP2; Sigma, Cat #M2320, AB_609904) were used. Both markers were chosen as they are often combined to capture all neuronal processes (Paik et al. 2019), and they have consistently been used as markers of differentiation in experiments with SH-SY5Y cells (Jahn et al. 2017; Shipley et al. 2016; Encinas et al. 2000; Kovalevich and Langford 2013; Paik et al. 2019). Following primary antibody incubation, cells were washed in alternating PBS and PBS-Tween 20 and incubated with secondary antibodies donkey anti-rabbit Alexa 488 (Invitrogen, Cat #A-21206 RRID:AB_141708), donkey anti-mouse Alexa 594 (Invitrogen, Cat #A-21203, RRID:AB_141633), and cell nuclei were stained with DAPI (CarlRoth, Cat #6843.3). Following secondary antibody incubation, coverslips were washed in cold PBS and mounted on glass microscope slides. Fluorescence imaging was done with the Olympus BX51WI microscope and disc spinning unit. Pictures were taken using the 20X objective lens. Micro-Manager software (Edelstein et al. 2014)(RRID:SCR_016865) was used to collect images. Further details on the primary and secondary antibody dilutions as well as microscope exposure times can be found in the Supplementary Material (Table S1 and S2).

### qRT-PCR

Cells in 6-well plates were first rinsed with PBS at 37 °C and then kept on ice for the rest of the extraction. RNA was extracted with TRIzol (Invitrogen, 15,596,026) according to the manufacturer’s protocol. RNA concentration was determined using the NanoDrop™ spectrophotometer, and cDNA was synthesized using RevertAid H Minus First Strand cDNA Synthesis Kit (Thermo Scientific, K1632). RNA was stored at −80 °C and cDNA at −20 °C. Three cell culture replicates were collected per time point, per condition.

Primers for qPCR were designed using NCBI gene reference database and Primer-BLAST (National Library of Medicine). The following primers were analyzed: Activity Regulated Cytoskeleton associated protein (*ARC,*Gene ID: 23,237), Early Response 1 (*EGR1*, Gene ID: 1958), cAMP Responsive Element Binding Protein 1 (*CREB1,* Gene ID: 1385), B-cell lymphoma 2 (*BCL2*, Gene ID: 596), BCL2- Associated X (*BAX,* Gene ID: 581), Brain Derived Neurotrophic Factor (*BDNF*, Gene ID: 627), Neurotrophic Receptor Tyrosine Kinase 2 (*NTRK2,* GeneID: 4915), Discs Large MAGUK scaffold protein 4 (*DLG4* also known as *PSD95,* GeneID:1742), Synaptophysin (*SYP,* GeneID:6855)*,* in conjunction with three House Keeping Genes (HKG’s): Glyceraldehyde-3-Phosphate Dehydrogenase (*GAPDH,* GeneID:2597), TATA-box Binding Protein (*TBP*,GeneID:6908), Peptidylprolyl Isomerase B (*PPiB*, GeneID:5479)*.* Primer sequences can be found in Supplementary Material Table S3. Primers at 600 nM concentration were mixed with Fast Start Universal Sybr Green Master ROX (Roche,491,385,001). Samples were run in 384-well qPCR plates (Roche,4TI-0382), using the LightCycler 480 Real-Time PCR system (Roche Life Sciences). qPCR program details are described in Supplementary Material Table S4.

## Analysis

### Microscopy

Image processing and analysis was done in Fiji (ImageJ version 1.52i, RRID:SCR_002285) (Schindelin et al. 2012). A DAPI nucleus staining was used to count total cells in each image. Neurite length and branching was measured in the 488 (βIII-Tubulin) channel, using the segmented line tool at 20X magnification. Per condition 70–100 neurites were measured. Typically, in differentiated SH-SY5Y cultures, many cells have only one or no neurites. Therefore, from each cell, we measured the length of the primary neurite, defined as the single neurite, or the longest neurite for cells having more than one neurite. An example of tracings of primary neurites as well as cells without neurite extensions can be seen in Fig. 2a. The NeuronJ plugin (Meijering et al. 2004) was used to quantify neurite length and neurite branching. For each image, primary neurites and branches were semi-automatically traced, and manually labeled as either primary neurites, or branches. The number of branches were divided by the total neurons (counted with DAPI), to give the number of branches per neuron in each image. An example neuron with branching can be seen in Fig. 2b. To identify whether the proportion of total cells with a primary extension changes due to serum deprivation, the number of primary neurites was also divided by total number of neurons (as counted with DAPI).

Total fluorescence of βIII-Tubulin staining was quantified using the 488 channel. First, the fluorescence threshold was set with the minimum intensity as the maximum background intensity. The total fluorescence intensity in the image was then measured, and corrected for cell area by dividing by the total area of cells in the 488 channel.

βIII-Tubulin stained cells were counted manually by setting the brightness contrast settings to 834 (min) and 7474 (max). Cells with visible green neurites were counted. MAP2 was manually counted in the 594 channel, at brightness contrast settings of 596 (min) and 6007 (max). These cell counts were divided by the total cells to calculate the proportion of βIII Tubulin or MAP2 cells.

### Gene Expression

A standard curve was used to calculate relative concentrations of gene expression per gene. An average of technical duplicates was made, and normalized to the average of 3 HKGs (TBP, PPiB, GAPDH). Analyses were performed with LightCycler 480 software version 1.5.1.62 (Roche Life Sciences) and Excel.

### Statistics

Statistical analysis and graphs were made with Prism 5 (Graphpad Software, USA, RRID:SCR_002798) and IBM Statistics 24 (SPSS for windows version 24.0, Armonk, NY:IBM Corp). Data collected from 2 independent experiments were pooled for statistical analysis. This resulted in a total of 4–14 images per condition being included in statistical analysis. For analysis of neurite length, an average neurite length per image was calculated from 70 to 100 neurites, for a total of 8 images per condition included in the 2-way ANOVA. For the analysis of primary neurites per neuron and neurite branching per neuron, the number of primary neurites in an image (between 30 and 100 per image) were divided by the total neurons in the image (between 210 and 250 cells per image), with 4–6 images per condition included in the 2-way ANOVA. For analysis of βIII-Tubulin Immunoreactivity, a total of 10–14 images per condition were included in the 2-way ANOVA. A 2-way ANOVA with factors Serum (serum, no serum) and Time (1,3,6, 24 h) was used for all comparisons of microscopy quantification and HKG normalized expression values. Bonferroni-corrected post-hoc tests were done in the case of significant interaction events. Reported results are mean ± standard error of the mean. Figures show bar graphs of the HKG normalized mean expression values; error bars are standard errors of the mean.

## Results

### Differentiation

**Fig. 1:**
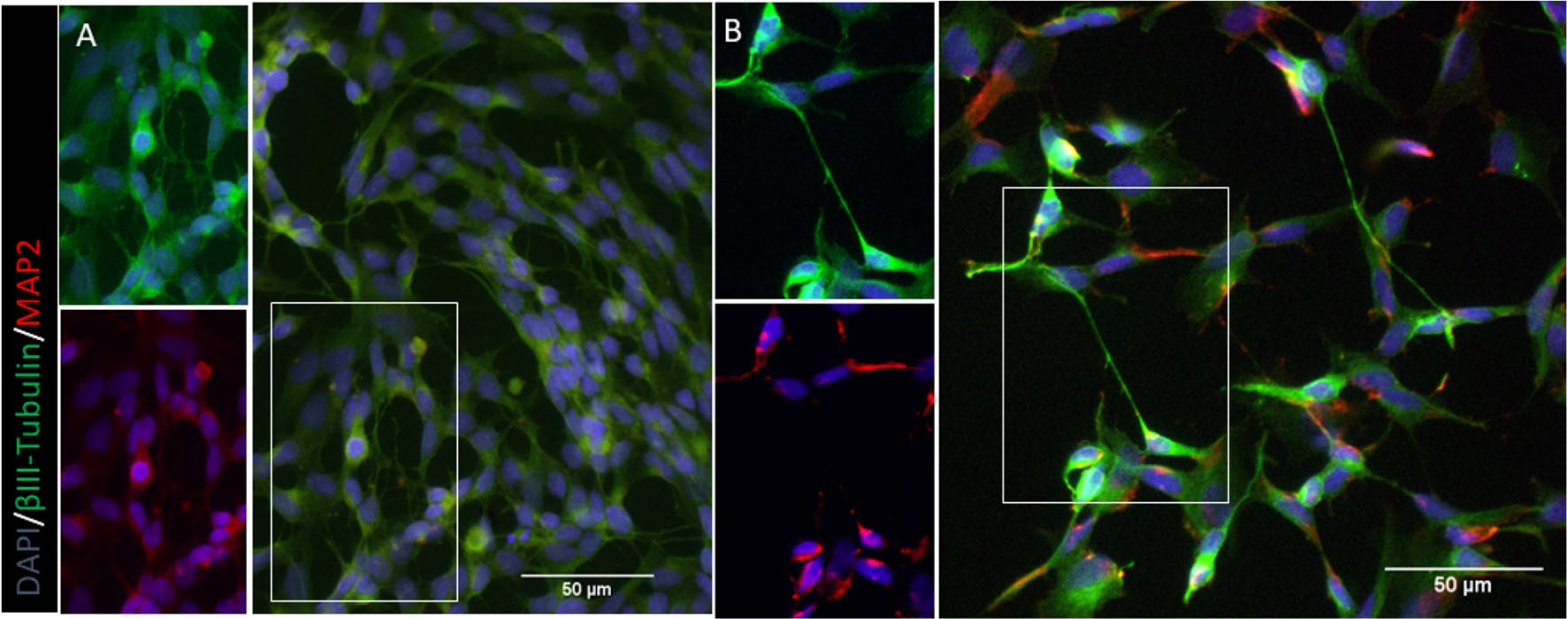
Visual representation SH-SY5Y cells. **a** Undifferentiated cells. **b** Neuron-like cells at 10 days differentiation

Differentiation was verified as explained previously (Thomson et al. 2020). Representative images comparing undifferentiated and differentiated cells can be seen in Fig. 1.

### Microscopy

**Fig. 2:**
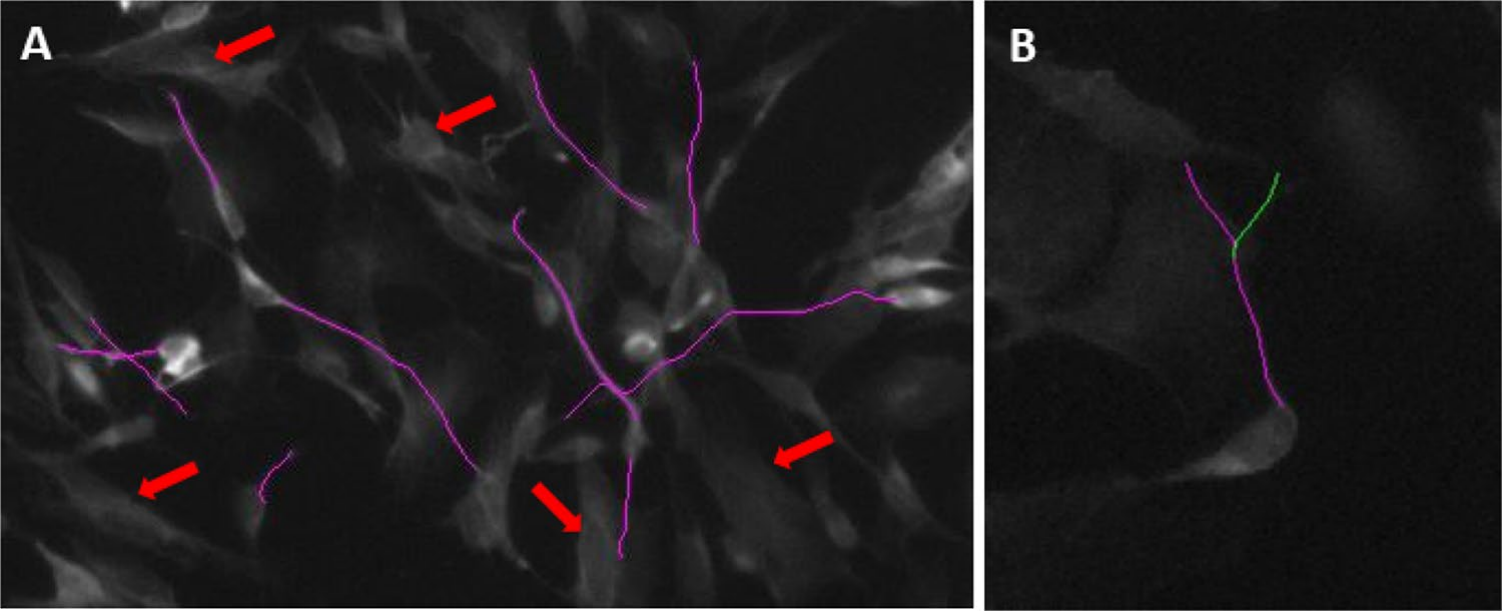
Morphological parameters analyzed. **a** Tracing of primary neurite extension in purple. An example of cells showing no neurite extensions are indicated with red arrows. **b** An example neuron tracing with branch. Primary neurite is traced in purple, branch is traced in green

Full statistical results of main effects (Time, Serum) and Time x Serum interaction effects for each parameter (Neurite Length, Neurite Branching, Primary Neurites, βIII-Tubulin immunoreactivity, and βIII-Tubulin and MAP2 positive cells) can be found in Supplementary Material Table S5. In case of significant main or interaction effects, the p-value of Bonferroni-corrected post-hoc tests are reported below. An example of the parameters measured can be seen in Fig. 2. An example of the morphology of cells immediately following serum removal can be seen in Supplementary Fig. 2.

#### Neurite Length

**Fig. 3:**
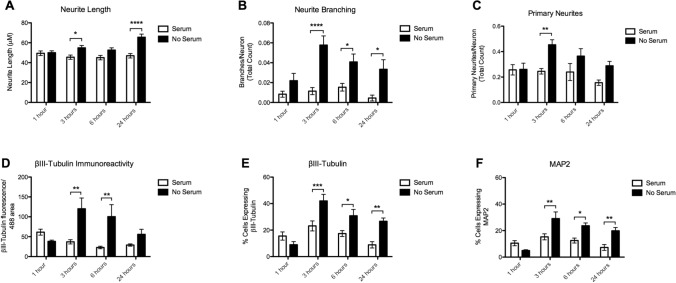
Morphological Parameters. **a** Neurite Length **b** Neurite Branching **c**. Primary Neurites **d** βIII-Tubulin immunoreactivity **e** Proportion βIII-Tubulin positive cells **f** Proportion MAP2 positive cells (Significant post hoc comparisons are indicated as *p < 0.05, **p < 0.01, ***p < 0.001, ****p < 0.0001)

We found a significant effect of Serum (*p* < 0.0001), but no significant effect of Time (*p* = 0.382), or Time x Serum interaction (*p* = 0.338) on neurite length. Serum deprived neurons had significantly longer outgrowths than neurons with serum at 3 h (54.93 ± 20.22 μm vs. 45.43 ± 17.76 μm, *p* = 0.03) and at 24 h (65.53 ± 27.74 μm vs. 46.89 ± 18.24 μm, *p* < 0.0001) (Fig. 3a).

#### Neurite Branching

There was a significant effect of Time (*p* = 0.0105) and Serum (*p* < 0.0001), but not a significant Time × Serum interaction (*p* = 0.062) on neurite branching. Serum deprived neurons had significantly more branches per neuron at 3 h (0.058 ± 0.008 vs. 0.011 ± 0.003, *p* < 0.0001), 6 h (0.041 ± 0.007 vs. 0.015 ± 0.003, *p* < 0.028), and at 24 h (0.033 ± 0.008 vs. 0.0045 ± 0.003, *p* < 0.028) (Fig. 3b).

#### Primary Neurites

Similarly, we found a significant effect of Time (*p* = 0.0368), Serum (*p* = 0.0006), but not a significant Time x Serum interaction (*p* = 0.109). At 3 h, serum deprived neurons showed a greater proportion with a primary neurite (0.45 ± 0.034 vs 0.25 ± 0.020, *p* = 0.001) (Fig. 3c).

#### βIII-Tubulin Immunoreactivity

A significant effect of Serum (*p* = 0.0006) and a Time x Serum interaction (*p* = 0.0035) was found, but no effect of Time (*p* = 0.14) on βIII-Tubulin immunoreactivity. There was a significant increase in βIII-Tubulin immunoreactivity in the serum deprived cells at 3 h (120.23 ± 25.58 vs. 37.03 ± 4.96, *p* < 0.01) and 6 h (110.49 ± 28.55 vs. 22.94 ± 3.03, *p* < 0.01) (Fig. 3d).

#### βIII-Tubulin and MAP2 Positive Cells

There was a significant effect of Time (p < 0.0001), Serum (*p* < 0.0001), and a Time × Serum interaction (*p* = 0.0012), on cells expressing βIII-Tubulin after serum deprivation. Serum deprived neurons showed a significant increase in the percentage of βIII-Tubulin expressing cells at 3 h (42.04 ± 4.92% vs. 23.16 ± 3.78%, *p* = 0.009) and 6 h (30.80 ± 2.77% vs. 17.40 ± 2.21%, *p* = 0.023), and 24 h (26.69 ± 2.41 vs. 8.90 ± 2.35, *p* = 0.00012) (Fig. 3e).

There was also a significant effect of Time (*p* < 0.0001), Serum (*p* < 0.0001), and a Time x Serum interaction (*p* = 0.0011), on percentage of cells expressing MAP2 after serum deprivation. Serum deprived cells also showed a significant increase in percentage MAP2 expressing cells at 3 h (29.08 ± 5.02% vs. 15.30 ± 2.28%, *p* < 0.01), 6 h (23.65 ± 2.12% vs. 12.51 ± 1.87%, *p* < 0.05), and 24 h (19.85 ± 2.50 vs. 7.27 ± 2.21, *p* < 0.01) (Fig. 3f).

### Gene Expression

We were most interested in gene expression changes following serum deprivation, specifically in genes related to IEG expression (*ARC, EGR1*), apoptosis (*BCL2*, *BAX*), plasticity (*BDNF, NTRK2*, *CREB1*) and synaptogenesis (*PSD95*, *SYP*). Full statistical results of main (Time, Serum) and Time x Serum interaction effects can be found in Supplementary Material Table S6. In case of significant main or interaction effects, p values and Bonferroni-corrected post hoc tests are reported in the text. Graphs show mean HKG normalized expression levels for each condition, error bars are standard error of the mean.

#### Immediate Early Gene Expression

We measured the expression of IEG’s *ARC* and *EGR1*, finding high expression levels in both genes in the 1 h serum condition only. Expression levels were low in all other samples (3 h, 6 h and 24 h), many of which were too low to detect (Ct value ≥ 34). Gene expression results can be found in Supplementary Material and Supplementary Fig. 1.

##### Apoptosis Markers

##### BCL2 Expression

**Fig. 4:**
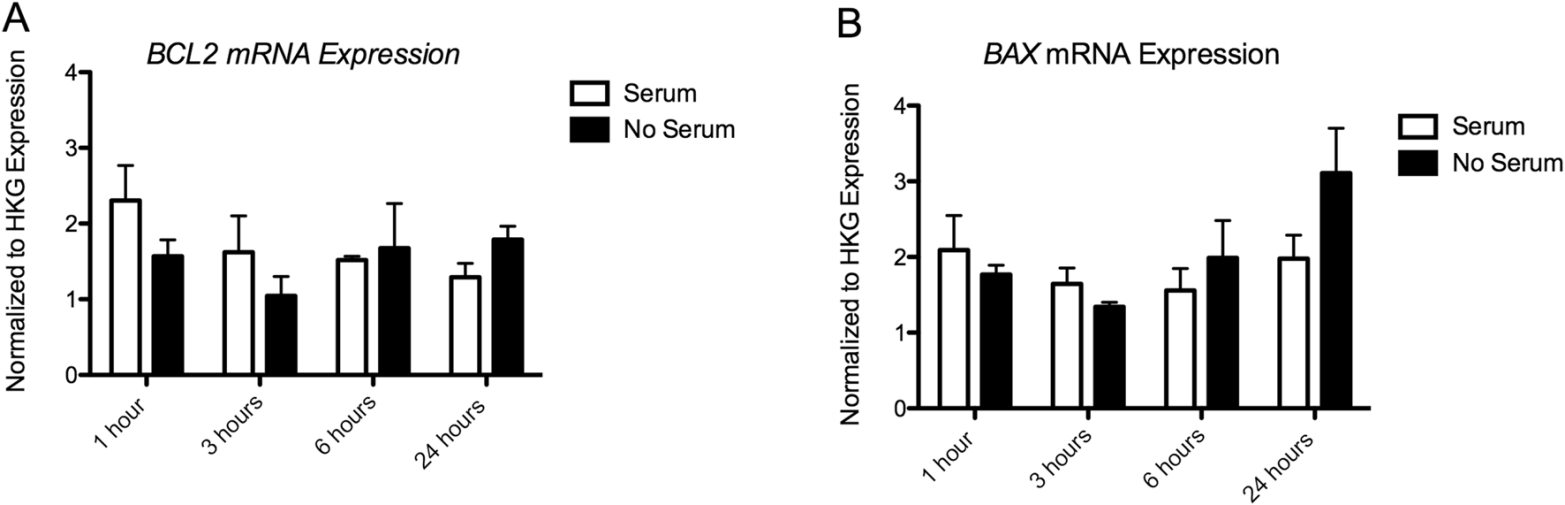
Results of gene expression analysis of **a.** *BCL2* mRNA and **b.** *BAX* mRNA. Expression values have been normalized to the average of 3 housekeeping genes (*TBP, PPiB, GAPDH*)

We also found no significant main effects of Time (*p* = 0. 362), Serum (*p* = 0.618), or Time × Serum interaction (*p* = 0.216) on BCL2 expression (Fig. 4a).

##### BAX Expression

We found no significant main effect of Time (*p* = 0.169), Serum (*p* = 0.380), or Time × Serum interaction (*p* = 0.228) on BAX expression (Fig. 4b)*.*

#### Expression of BDNF Signaling

##### BDNF Expression

**Fig. 5:**
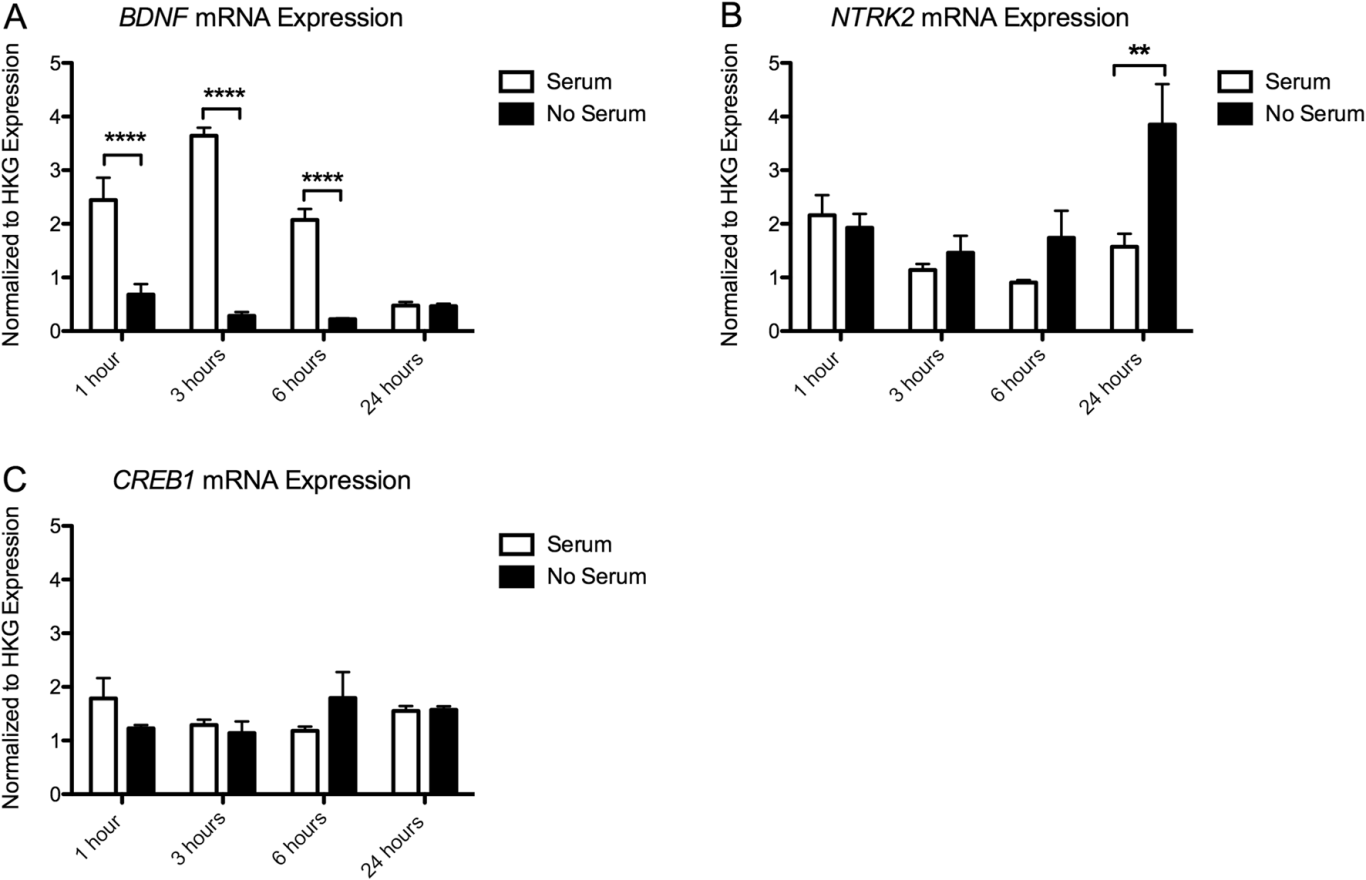
Results of gene expression analysis of **a.** *BDNF* mRNA and **b.** *NTRK2* mRNA, and **c. CREB** mRNA. Expression values have been normalized to the average of 3 housekeeping genes (*TBP, PPiB, GAPDH*). Significant post hoc comparisons are indicated as, **p < 0.01, ****p < 0.0001

We found a significant effect of Time (*p* < 0.001), Serum (*p* < 0.001) and Time × Serum interaction (*p* < 0.001) on *BDNF* expression. There was a significant decrease in *BDNF* expression in the serum deprived cells at 1 h (*p* < 0.0001), 3 h (*p* < 0.0001), and 6 h (*p* < 0.0001). Compared to time-matched serum controls, serum deprived cells express 25.71 ± 5.76% *BDNF* at 1 h, 8.07 ± 1.35% at 3 h, 12.01 ± 0.61% at 6 h and 94.58 ± 5.89% at 24 h (Fig. 5a).

##### NTRK2 Expression

There was also a significant effect of Time (*p* = 0. 008), Serum (*p* = 0.006), and Time × Serum interaction (*p* = 0.021) on *NTRK2* expression. There was a significant increase in expression of *NTRK2* mRNA at 24 h (*p* < 0.01), with serum deprived cells expressing 242.11 ± 33.00% of the *NTRK2* expressed in serum controls (Fig. 5b).

##### CREB Expression

There was no significant main effect of Time (*p* = 0. 393) or Serum (*p* = 0.942), on CREB expression. However, there was a slight Time × Serum interaction effect (*p* = 0.043). Initially, there is a decrease in *CREB* expression in serum deprived cells at 1 h (to 65.25 ± 1.26% serum controls), at 6 h this is reversed (164.71 ± 30.31% serum controls) (Fig. 5c). None of these time points are significant in Bonferroni-corrected post hoc tests.

#### Synaptogenesis Genes

##### PSD95 Expression

**Fig. 6:**
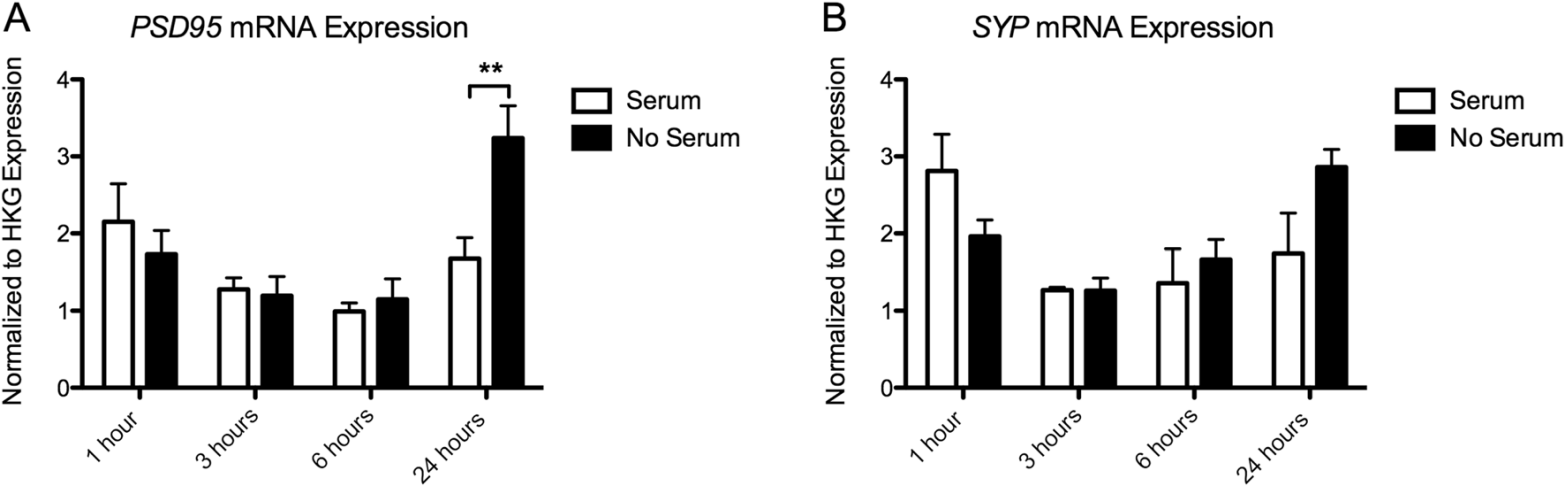
Gene expression analysis of A.*PSD95* mRNA and B. *SYP* mRNA. Expression values have been normalized to the average of 3 housekeeping genes (TBP, PPiB, GAPDH). Significant post hoc comparisons are indicated as, **p < 0.01

There was a significant effect of Time (*p* = 0.001), and a Time × Serum interaction (*p* = 0.020), but no effect of Serum (*p* = 0.153), on *PSD95* expression. There was a significant increase in expression in the serum-deprived cells at 24 h (*p* < 0.01).Serum-deprived cells express 192.85 ± 17.78% of the serum controls at 24 h (Fig. 6a).

##### SYP Expression

Again, we found a significant effect of Time (*p* = 0.017), but no effect of Serum (*p* = 0.575). We found a trend towards a significant Time × Serum interaction effect (*p* = 0.059). (Fig. 6b).

## Discussion

In this study, we aimed to systematically characterize, in fully differentiated SH-SY5Y cells, the effects of complete serum removal on several morphological and gene expression markers of plasticity. We found that serum removal, over 24 h, increased primary neurite length as well as neurite branching when compared to serum controls. Serum deprived neurons also showed higher levels of βIII-Tubulin immunoreactivity, and a greater proportion of βIII-Tubulin- and MAP2-positive neurons. MAP2 is mainly localized in mature dendrites (Dehmelt and Halpain 2005), and βIII-Tubulin is a widely used neuronal maturity marker (Katsetos et al. 1993). These findings suggest that in fully differentiated cells, complete serum removal may promote additional plasticity-like effects. This can be seen as early as 3 h following removal of serum, and lasts at least 24 h.

We also found that complete serum removal has a specific effect on the expression of several genes involved in BDNF-TrkB signaling and synaptogenesis. Serum deprivation resulted in a significant increase in the expression of *NTRK2, PSD95* and *SYP* over time, with the strongest effect on the expression of *NTRK2* and *PSD95* mRNA at 24 h following deprivation. *NTRK2*, the gene coding for the TrkB receptor, has been shown to be important in activity dependent plasticity leading to long term potentiation (LTP) (Minichiello 2009; Minichiello et al. 1999). PSD-95 is an important scaffolding protein, regulating the strength of excitatory synapses (Chen et al. 2015), and the *SYP* gene codes for synaptophysin, an important protein involved in neurotransmitter release (Südhof et al. 1987; Arthur and Stowell 2007). An increase in the expression of *NTRK2, PSD95* and *SYP* mRNA over time in serum-deprived cells therefore aligns with our morphology results. Our results suggest that complete serum removal induces an increased expression of genes and morphological markers of plasticity and synaptic strength, potentially confounding experiments interested in these outcome measures.

While these observations are in line with the differentiation-inducing effect of serum deprivation (Encinas et al. 2000; Jahn et al. 2017; Shipley et al. 2016; Kaplan et al. 1993), these cells are already fully differentiated, therefore the additional changes in morphological markers that we present here may indicate additional, confounding plasticity effects. Indeed, systematic transcriptomic profiling SH-SY5Y cells has identified *NTRK2* as well as many genes involved in neurogenesis and cytoskeletal reorganization as upregulated in differentiated compared to undifferentiated cells (Pezzini et al. 2017). However, once cells have been differentiated, the expression of these genes is stable over time; in contrast to the serum removal effects we report here. This semi-acute increase in plasticity-related gene expression and morphological markers is problematic in studies using these genes or morphological plasticity markers as outcome measures.

Interestingly, we also report a strong effect of serum on the expression of *BDNF, ARC* and *EGR1*. *BDNF* expression increased in the in the serum control cells after 1, 3 and 6 h, returning to low expression levels at 24 h. The serum control cells underwent a regular medium change, including a PBS wash step. This increase in *BDNF* mRNA in the serum condition is surprising, but may be related to the addition of fresh medium and serum. In serum-deprived cells, this temporary increase is absent, likely due to the disruption of growth and protein production as a consequence of serum withdrawal (Inoue et al. 1996; Irie et al. 1999; Ozturk et al. 2003; Ozturk and Palsson 1991). We also report an increase in expression of the immediate early genes *ARC* and *EGR1* in the serum condition at 1 h after PBS wash and serum replacement. The removal and re-addition of serum could have induced an immediate but transient increase in the expression of *ARC* and *EGR1* mRNA, in line with the expected expression pattern of an immediate early gene (Lyford et al. 1995; Schratt et al. 2001).

We did not find any effects of serum deprivation on the expression of genes linked to apoptosis, *BAX* and *BCL2*. Encinas et al. (2000) showed that SH-SY5Y cells show signs of apoptosis 6 and 24 h after serum removal as measured by caspase activity and TUNEL assay (Encinas et al. 2000). Encinas et al. (2000) used cells that were treated with RA for only five days and in medium containing 15% FBS. The shock of serum removal in the not-fully differentiated cells is likely much stronger compared to our protocol, and may explain the different finding. Based on the gene expression markers in our experiments, we cannot confirm that serum starvation influences apoptotic processes after 24 h in in fully differentiated SH-SY5Y cells.

## Conclusion

Despite being common practice to remove serum from the culture medium of already differentiated SH-SY5Y cultures before experimentation, the effects on morphology and gene expression had not been systematically characterized. Here, we show that complete serum deprivation has an effect on commonly used morphological and gene expression markers of cellular and synaptic plasticity in differentiated SH-SY5Y cells, and may thus confound results when examining plasticity-related outcome measures. For future research involving differentiated SH-SY5Y cells as a model of human neural plasticity, our findings provide some key considerations for experimental design. Studies interested in measuring plasticity effects in differentiated SH-SY5Y cells should either refrain from complete serum deprivation 24 h before experimentation, or include appropriate controls, e.g. cells which were not serum deprived, to confirm serum deprivation had no confounding effects on outcome measures.

## Supplementary Information

Below is the link to the electronic supplementary material.

Supplementary file1 (DOCX 709 KB)

## Data Availability

All data available upon reasonable request.

## Abbreviations

FBS: Fetal Bovine Serum
RA: Retinoic Acid
MAP2: Microtubule Associated Protein 2
ARC: Activity Regulated Cytoskeleton associated protein
EGR1: Early Growth Response 1
BCL2: B-cell Lymphoma 2
BAX: BCL2- Associated X
BDNF: Brain-Derived Neurotrophic Factor
NTRK2: Neurotrophic Receptor Tyrosine Kinase 2
CREB1: CAMP Responsive Element Binding Protein 1
PSD95: Discs Large MAGUK scaffold protein 4, DLG4
SYP: Synaptophysin
TBP: TATA-Box Binding Protein
GAPDH: Glyceraldehyde 3-Phosphate Dehydrogenase
PPiB: Peptidylprolyl Isomerase B
