# Supplementary material for: The Effects of Serum Removal on Gene Expression and Morphological Plasticity Markers in Differentiated SH-SY5Y Cells
Source: Cell Mol Neurobiol. 2021 Mar 3;42(6):1829–39. doi: 10.1007/s10571-021-01062-x (PMC9239930; doi:10.1007/s10571-021-01062-x)
Supplement: Supplementary file 1 — Supplementary file1 (DOCX 709 KB) [file 10571_2021_1062_MOESM1_ESM.docx]

| **Excitation/Emission** | **Marker of** | **Exposure Time (ms)** | **Color** |
| --- | --- | --- | --- |
| 358/461 | DAPI (nuclei) | 10 | Blue |
| 493/519 | βIII-Tubulin | 500 | Green |
| 591/614 | MAP2 | 300 | Red |

Supplementary Material

**Table S1.** Exposure times for fluorescence microscopy. Olympus BX51WI microscope and DSU spinning unit used. Pictures were taken using the 20X objective lens.

| **Name** | **Company** | **Order number** | **Concentration used** | **Marker of** |
| --- | --- | --- | --- | --- |
| βIII Tubulin | Cell Signaling | 5568S | 1:300 | Neurons;  Axon guidance and maturation |
| MAP2 | Sigma | M2320 | 1:300 | Neurons;  Dendrite formation |
| Donkey anti rabbit Alexa 488 | Invitrogen | A21206 | 1:1000 | βIII Tubulin |
| Donkey anti mouse Alexa 594 | Invitrogen | A21203 | 1:1000 | MAP2 |
| DAPI | CarlRoth | 6843.3 | 1:800 | Nucleus |

**Table S2.** Primary and secondary antibody dilutions used for immunocytochemistry.

| *GAPDH* | FWD | CCAAATGCGTTGACTCCGA |
| --- | --- | --- |
| *GAPDH* | REV | GCATCTTCTTTTGCGTCGC​ |
| *TBP* | FWD | TGCACAGGAGCCAAGAGTGAA |
| *TBP* | REV | CACATCACAGCTCCCCACCA |
| *PPiB* | FWD | GTTTGAAGTTCTCATCGGGG |
| *PPiB* | REV | AAAACAGCAAATTCCATCGTG |
| *ARC* | FWD | GGAGTACTGGCTGTCCCAGA |
| *ARC* | REV | ACTCCACCCAGTTCTTCACG |
| *EGR1* | FWD | CCCCGACTACCTGTTTCCAC |
| *EGR1* | REV | GACAGAGGGGTTAGCGAAGG |
| *CREB1* | FWD | CCCCAGCACTTCCTACACAG |
| *CREB1* | REV | CTCGAGCTGCTTCCCTGTTC |
| *BCL2* | FWD | ACATCGCCCTGTGGATGACT |
| *BCL2* | REV | CCGTACAGTTCCACAAAGGC |
| *BAX* | FWD | GGGGACGAACTGGACAGTAA |
| *BAX* | REV | CAGTTGAAGTTGCCGTCAGA |
| *BDNF* | FWD | ATAGAGTGTGGGAGTTTTGGGG |
| *BDNF* | REV | TGGTGGAACTTTTCAGTCACTACT |
| *NTRK2* | FWD | TGGATGCATATCGTGCTCCG |
| *NTRK2* | REV | GTGCTTGGTTCAGCTCTTGC |
| *PSD95* | FWD | CCCCAGGATATGTGAACGGG |
| *PSD95* | REV | CCGATGTGTGGGTTGTCAGT |
| *SYP* | FWD | CACTGATGACTTCCCAGAACTGT |
| *SYP* | REV | CTGGGCTTCACTGACCAGAC |

**Table S3**. Primer Sequences, 5’ to 3’ orientation. All primers were designed using the NCBI gene reference database and Primer-BLAST (National Library of Medicine, https://www.ncbi.nlm.nih.gov/tools/primer-blast/). Resulting primer sequences were crosschecked with the CCDS sequence for overlap and primer suitability.

| **Analysis Mode** | **Program Name** | **cycles** | Target (°C) | Acquisition Mode | Hold (hh:mm:ss) | Ramp Rate (°C/s) |
| --- | --- | --- | --- | --- | --- | --- |
| None | Pre Incubation | 1 | 95 | None | 0:10:00 | 4.8 |
| Quantification | Amplification | 45 | 95 | None | 0:00:10 | 4.8 |
|  |  |  | 60 | single | 0:00:45 | 2.5 |
| Melting Curves | Melting Curve | 1 | 95 | None | 0:00:15 | 4.8 |
|  |  |  | 60 | None | 0:00:30 | 2.5 |
|  |  |  | 97 | Continuous |  | 0.11 |
| None | Cooling | 1 | 60 | None | 0:00:10 | 2.5 |

**Table S4.** qRT-PCR program. Run in 384 well qPCR plates (Roche,4TI-0382) using LightCycler® 480 Real-Time PCR (Roche LifeScience).

**Full Statistical Results**

| Parameter | Time | Serum | Time x Serum Interaction |
| --- | --- | --- | --- |
| Neurite Length | *F*(3,55)=1.041  p=0.3819 | ***F*(1,55)=22.33 p<0.0001** | *F*(3,55)=1.148  p=0.3380 |
| Neurite Branching | ***F*(3,29)=4.482**  **p=0.0105** | ***F*(1,29)=43.45**  **p<0.0001** | *F*(3,29)=2.727  p=0.0622 |
| Primary Neurites | ***F*(3,29)=3.229**  **p=0.0368** | ***F*(1,29)=14.81**  **p=0.0006** | *F*(3,29)=2.203  p=0.109 |
| βIII-Tubulin Immunoreactivity | *F*(3,73)=1.856  p=0.14 | ***F*(1,73)= 13.04**  **p=0.0006** | ***F*(3,73)= 4.943**  **p=0.0035** |
| βIII-Tubulin positive cells | ***F*(3,56)=13.20**  **p<0.0001** | ***F*(1,56)=20.46**  **p<0.0001** | ***F*(3,56)=6.083**  **p=0.0012** |
| MAP2 positive cells | ***F*(3,56)=11.48**  **p<0.0001** | ***F*(1,56)=19.09, p<0.0001** | ***F*(3,56)=6.151**  **p=0.0011** |

**Table S5.** Morphological parameters main and interaction effects. Significant main effects in bold.

| Gene | Time | Serum | Time x Serum Interaction |
| --- | --- | --- | --- |
| *BCL2* | *F*(3,16)=1.144  p=0. 362 | *F*(1,16)=0.258  p=0.618 | *F*(3,16)= 1.659  p=0.216 |
| *BAX* | *F*(3,16)=1.906  p=0.169 | *F*(1,16)=0.816  p=0.380 | *F*(3,16)=1.602  p=0.228 |
| *BDNF* | ***F*(3,15)=16.250 p<0.001** | ***F*(1,15)=128.71 p<0.001** | ***F*(3,15)=17.547 p<0.001** |
| *NTRK2* | ***F*(3,16)=5.650**  **p=0.008** | ***F*(1,16)=9.791**  **p=0.006** | ***F*(3,16)= 4.300**  **p=0.021** |
| *CREB1* | *F*(3,16)=1.063  p=0. 393 | *F*(1,16)=0.005  p=0.942 | ***F*(3,16)= 3.403**  **p=0. 043** |
| *PSD95* | ***F*(3,16)=8.338**  **p=0.001** | *F*(1,16)=2.256  p=0.153 | ***F*(3,16)= 4.339**  **p=0.020** |
| *SYP* | ***F*(3,16)=4.550**  **p=0.017** | *F*(1,16)=0.327  p=0.575 | *F*(3,16)= 3.054  p=0.059 |

**Table S6.** Gene expression main and interaction effect results. Significant main effects in bold.

**Immediate Early Gene Expression Results**

| Gene | Time | Serum | Time x Serum Interaction |
| --- | --- | --- | --- |
| *ARC* | ***F*(3,16)=94.119 p<0.0001** | ***F*(1,16)=127.846 p<0.0001** | ***F*(3,16)=90.539 p<0.0001** |
| *EGR1* | ***F*(3,16)=21.815 p<0.0001** | ***F*(1,16)=25.455 p<0.0001** | ***F*(3,16)=22.007 p<0.0001** |

***ARC Expression:*** We found a significant main effect of Time, Serum, and a Time x Serum interaction on *ARC* expression. Post-hoc analysis showed that cells that were serum deprived show a significant decrease in HKG normalized *ARC* expression compared to serum cells at 1 hour (p<0.0001). When normalized to the *ARC* expression in serum time-matched controls, serum deprived cells expressed 3.23±0.40% of the *ARC* mRNA at 1 hour, 11.76±1.60% at 3 hours, 76.74±26.40% at 6 hours, and 97.38±10.89% at 24 hours (Supplementary Figure 1A).

***EGR1 Expression:*** Again, we found a significant main effect of Time, Serum, and a Time x Serum interaction on *EGR1* expression. Post-hoc analysis showed a significant decrease in expression in the serum-deprived cells at 1 hour (p<0.001). Compared to the serum condition, *EGR1* expression in serum-deprived cells was 0.92±0.04% at 1hour, 5.18±0.60% at 3 hours, 54.10±10.03% at 6 hours, and 167.93±61.40% at 24 hours (Supplementary Figure 1B).


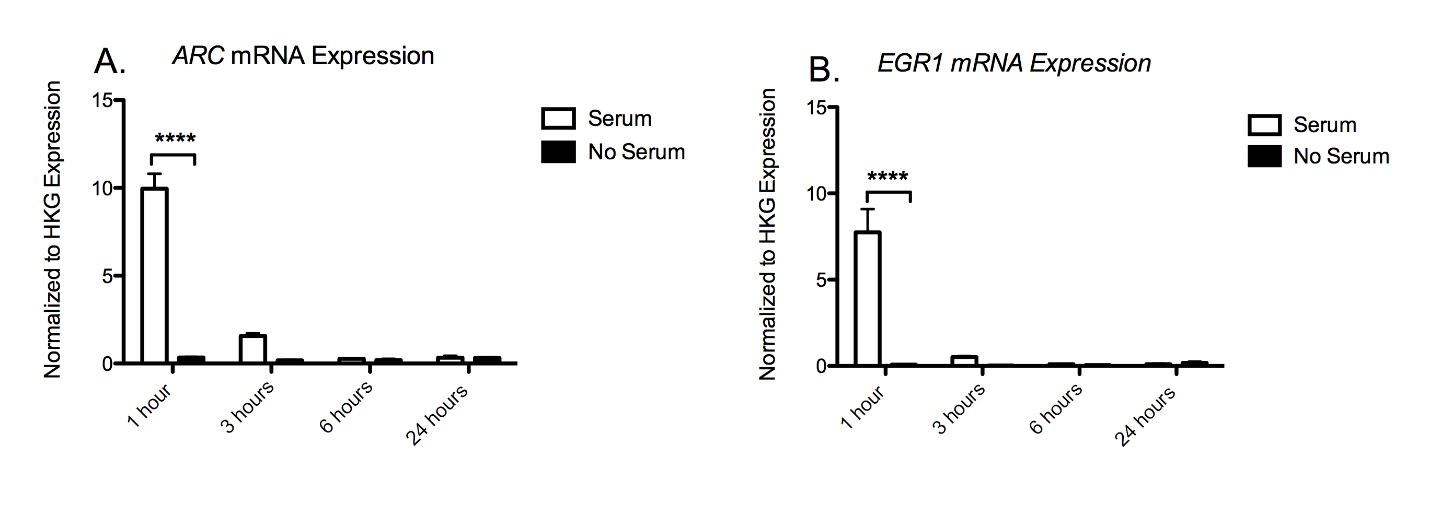


**Supplementary Figure 1.** Gene expression levels of **A.** *ARC* and **B.** *EGR1.* Expression levels are normalized to the average of 3 housekeeping genes. Significant post hoc comparisons are indicated ****P<0.0001.


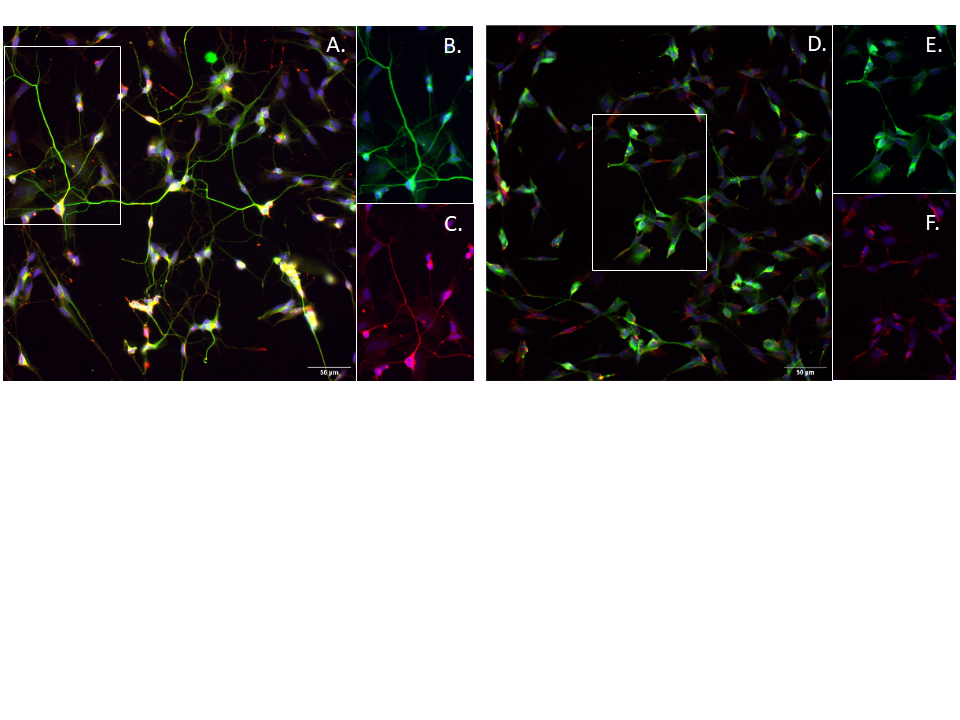


**Supplementary Figure 2.** **A.** Example of morphology of differentiated SH-SY5Y cells immediately following serum removal (**A-C**) compared to cells in serum-containing media (**D-F**). Nucleus (DAPI) in blue, βIII-Tubulin in green, MAP2 in red. **A.** Serum-deprived cells, merge of all channels, **B.** Serum-deprived cells, DAPI and βIII-Tubulin **C.** Serum-deprived cells, DAPI and MAP2 **D.** Cells in serum, merge of all channels **E.** Cells in serum, DAPI and βIII-Tubulin **F.** Cells in serum, DAPI and MAP2.
